# Tauopathies: new perspectives and challenges

**DOI:** 10.1186/s13024-022-00533-z

**Published:** 2022-04-07

**Authors:** Yi Zhang, Kai-Min Wu, Liu Yang, Qiang Dong, Jin-Tai Yu

**Affiliations:** Department of Neurology and Institute of Neurology, Huashan Hospital, State Key Laboratory of Medical Neurobiology and MOE Frontiers Center for Brain Science, Shanghai Medical College, Fudan University, National Center for Neurological Disorders, 12th Wulumuqi Zhong Road, Shanghai, 200040 China

**Keywords:** Tauopathies, Neurodegeneration, Biomarkers, Therapeutics, Genetics

## Abstract

**Background:**

Tauopathies are a class of neurodegenerative disorders characterized by neuronal and/or glial tau-positive inclusions.

**Main body:**

Clinically, tauopathies can present with a range of phenotypes that include cognitive/behavioral-disorders, movement disorders, language disorders and non-specific amnestic symptoms in advanced age. Pathologically, tauopathies can be classified based on the predominant tau isoforms that are present in the inclusion bodies (i.e., 3R, 4R or equal 3R:4R ratio). Imaging, cerebrospinal fluid (CSF) and blood-based tau biomarkers have the potential to be used as a routine diagnostic strategy and in the evaluation of patients with tauopathies. As tauopathies are strongly linked neuropathologically and genetically to tau protein abnormalities, there is a growing interest in pursuing of tau-directed therapeutics for the disorders. Here we synthesize emerging lessons on tauopathies from clinical, pathological, genetic, and experimental studies toward a unified concept of these disorders that may accelerate the therapeutics.

**Conclusions:**

Since tauopathies are still untreatable diseases, efforts have been made to depict clinical and pathological characteristics, identify biomarkers, elucidate underlying pathogenesis to achieve early diagnosis and develop disease-modifying therapies.

## Background

“Tauopathies” was coined as an umbrella word depicting some neurodegenerative disorders [1], which are characterized by tau deposits in the brain (mainly in neurons, also in glial cells and extracellular space), with symptoms of dementia and parkinsonism. Currently, more than 26 different tauopathies have been identified [2]. Depending on the major tau isoforms appearing in aggregates, tauopathies are usually classified into 3R tauopathies (mainly having 3R tau), 4R tauopathies (mainly having 4R tau) and 3R/4R tauopathies (with approximately an equal ratio of 3R tau and 4R tau). Besides, in primary tauopathies, tau is the major and prominent component of the pathology, such as PiD (Pick's disease), PSP (Progressive supranuclear palsy), CBD (corticobasal degeneration) and AGD (Argyrophilic grain disease). While in secondary tauopathies, tau aggregation is regarded as a response to other pathological proteins or events [2], like amyloid beta (Aβ) in AD (Alzheimer’s disease) and repetitive brain injury in CTE (chronic traumatic encephalopathy).

Tauopathies have varied symptoms and complicated manifestations and pathology overlap. Moreover, the mechanisms underlying neurodegeneration of tauopathies are intricate, among which pathological tau forms, tau aggregation and propagation may play important roles. Though risk for tauopathies is partially attributed to genetics, study of related genetic variants helps to understand the mechanisms of disease. Nowadays, biomarkers and preclinical studies greatly subserve the diagnosis and treatment of tauopathies. In this review, we summarize recent reports about epidemiology, clinical phenotypes, pathology, genetics and the development and challenges in tau related biomarkers and therapies.

## Main text

### Tau structure and function

**Fig. 1:**
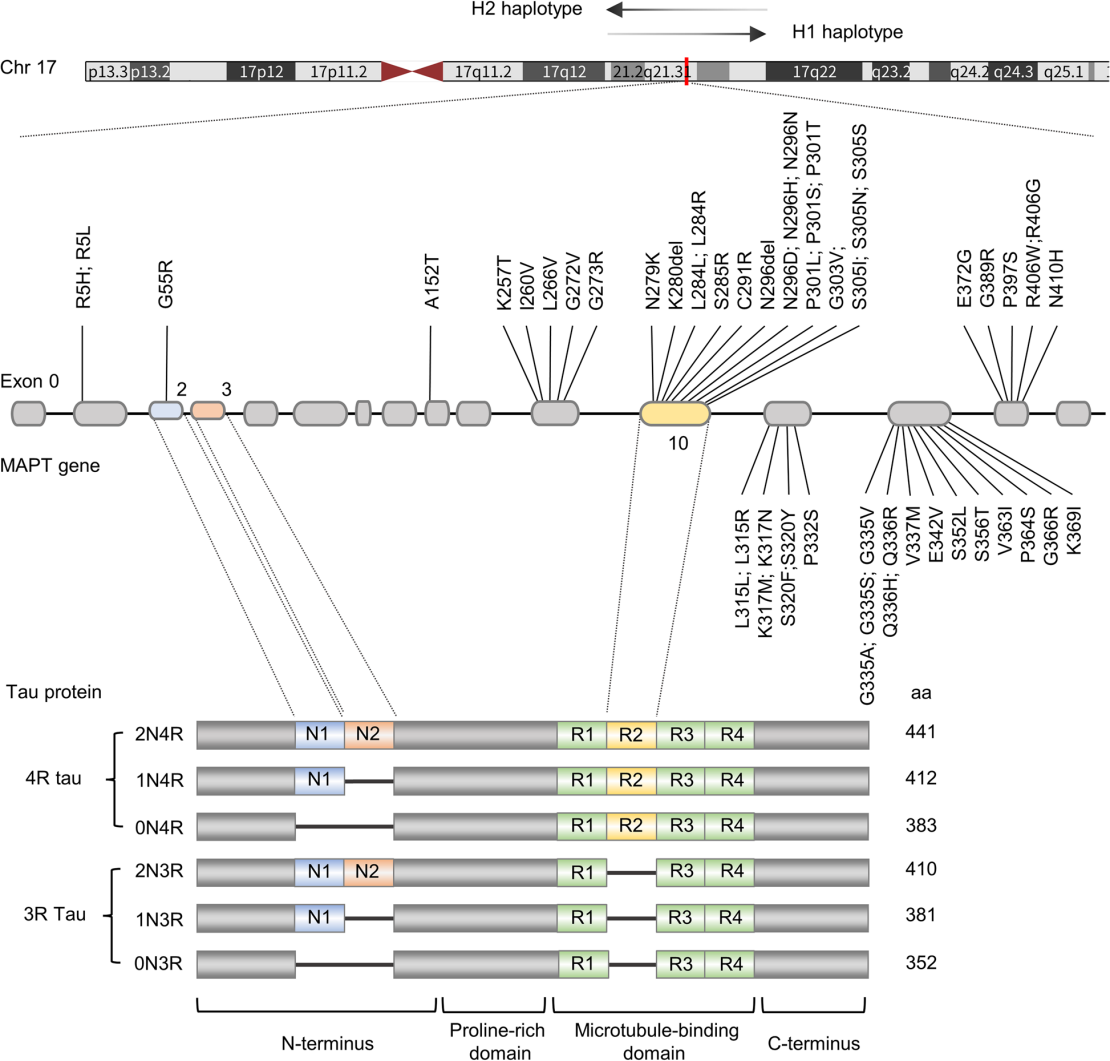
MAPT pathological mutations in exons, haplotypes, and alternative splicing. MAPT (microtubule associated protein tau) gene, located on chromosome 17q21.3, has been identified with over 100 mutations, and pathological mutations associated with increased risk for tauopathies are shown above. The difference between H1 and H2 haplotypes is a 900 kb inversion existing in the largest linkage disequilibrium (LD) area in chromosome 17. Besides, H1 and its various sub-haplotypes usually contribute to disease occurrence, while H2 haplotype often act as a protective factor. Alternative splicing is common in neuronal cells which help to increase genetic plasticity and the diversity of proteome under physiological conditions [6]. However, imbalance in the ratio of the 3R and 4R isoforms can give rise to the pathogenesis of tauopathies, as the 4R tau is more efficient in promoting microtubule assembly with an extra repeat domain R2 which hyper-stabilize MT and more free-floating tau leads to aggregates formation [3, 6, 7]

Tau is encoded by the microtubule associated protein tau (MAPT) gene, which is located on chromosome 17q21.3, containing 16 exons. In its precursor messenger RNA (pre-mRNA), alternative splicing of exon 2 (E2), E3 and E10 generates six tau isoforms, and the length range of tau protein is between 352 and 441 amino acids [1] (Fig. 1). Depending on the existence of three or four repeats of MTBDs (microtubule binding domains), tau can be classified into 3R tau (exon 10 exclusion) and 4R tau (exon 10 inclusion) [1, 3]. No isoform contains an isolated E3 without E2. The levels of the 3R and 4R isoforms are approximately equal in the adult human brain [4]. Another less discussed tau isoform is Big tau, which is also generated from alternative splicing of pre-mRNA from the MAPT gene. It has an extra E4a, leading to its higher molecular weight ~ 110 kDa. Due to its large size and few phosphorylated sites, Big tau was hypothesized to have a lower propensity to form pathological misfolding. This theory is in line with spared PNS (peripheral nervous system) in tauopathies, where Big tau is mainly expressed [5].

Tau mainly exists in the axons of neurons under physiological conditions [1]. The MTBDs bind to tubulins, promoting microtubule assembly and stability. In addition, tau knock-out mice developed glucose intolerance, pancreatic disorders, anxiety, and impairment of contextual and cued fear memory, implying a wide range of undiscovered functions of tau [8]. Tau also plays a role in protecting genomic architecture, regulating myelination and synaptic plasticity, iron homeostasis, and neurogenesis [9, 10]. Further scrutiny might influence anti-tau therapies as the physiological functions of tau are unintentionally disturbed by treatment.

Regulation of affinity between tau and microtubules mainly depends on phosphorylation of tau, which mostly happens in proline-rich region and C-terminus [11]. However, hyperphosphorylation at some sites will reduce binding affinity, resulting in instability of microtubules, also affecting axonal transport and neurotransmission [11]. Besides, other alterations of tau such as overexpression, mutations, other aberrant posttranslational modifications in addition to phosphorylation (acetylation, truncation, O-GlcNAcylation, etc.), abnormal ratio between tau isoforms and mis-localization could lead to pathological changes observed in tauopathies [10].

 The role of tau in neurodegeneration is still not elucidated. Mechanisms have been commonly summarized as loss of function, gain of function and mislocalization due to tau abnormality [10]. A recent study characterized tau interactomes in human induced pluripotent stem cell (iPSC)-derived neurons. They revealed the direct interactions between pathological tau and proteins, including SNARE complex, RNA-binding proteins, and mitochondrial proteins, which influenced tau release, protein synthesis and energy supply respectively. It helped to discover tau mediated pathogenesis and potential therapeutic strategies [12].

### Epidemiology of tauopathies

According to WHO, more than 55.2 million people worldwide currently suffer from dementia, with approximately 7 million new cases per year, and the data will increase to 139 million in 2050 [13]. Aging is still the greatest risk factor of all. A cross-sectional study recruiting 46,011 adults over 60 in China suggested a significantly higher prevalence of dementia in population with older age (OR: 2.69–6.60), female gender (OR: 1.43) and family history (OR: 7.20) [14]. Other modifiable risk factors include education, overall health [15], lifestyle, rural residence and environmental factors [16] like PM 2.5 [17] and transportation noise [18].

Frontotemporal dementia (FTD) account for 2.6% of all-cause dementia. Though regarded as the most common presenile dementia, the incidence of FTD increased over time and peaked at the age of 75–79 [19, 20]. The age-standardized incidence of FTD was 2.90 per 100,000 person-year. Time ranges of survival changed slightly among bvFTD (behavioral variant of frontotemporal dementia) and AD, whereas FTD patients initially developing motor symptoms (all having PSP or CBD as underlying pathology) had the shortest survival time [19]. Accuracy of data about FTD is hampered by underdiagnosis and different criteria adopted. For better estimation, a multicenter prospective observational study designed in Europe is underway [21].

In Olmsted County, the incidence of PSP and CBS (corticobasal syndrome) increased over time, being 2.6 per 100,000 person-years and 0.4 per 100,000 person-years respectively [22], while another study found similar prevalence between them (around10.84/100,000) [23]. Most of patients are diagnosed at the age of 78 (70–74 for PSP, 75–79 for CBS [23]) with median survival time around 6 years [22].

AD is the most common type of dementia, accounting for 60–70% of all cases globally [19]. From 2010–2012, the prevalence was 14.7%, which remained steady since 1994 and was higher in low-income countries. However, the annual incidence dropped prominently from 2.8% in 1998–2000 to 2.2% in 2010–2012 [24]. Another study observed a 13% decrease per decade over the past 25 years [25]. Additionally, in non-western countries, the incidence of AD rose significantly among groups aged between 65 and 74 [26].

AGD is commonly regarded as a late on-set disease with mixed pathology. In a non-Caucasian population, 15.2% of participants were diagnosed with AGD, whose mean age were 78.9 ± 9.4 years [27]. In younger population with potentially purer AGD pathology, a study identified 7 patients via postmortem examination, only 2 were female. Most patients died at 64 years old with median survival time being 3 months (0.5–36) [28].

The clinical and neuropathological diagnosis criteria of CTE [29] haven’t reached a consensus due to the small number of cases studied. Thus, the exact incidence of CTE is unknown [30]. It has been reported that CTE is unavoidable in some sports. One study found that CTE pathology was more common in athletes and was mainly observed in men [31]. American football was the sport most associated with CTE (OR: 2.62) [31]. Longer playing years or a higher level of professionalism were linked to an increased risk of CTE and pathology severity [32, 33]. Prospective, autopsy studies of populations from different exposure backgrounds are currently needed to confirm its epidemiology and public impacts [30, 31].

### Clinical symptoms

Tauopathies lead to a variety of behavioral, movement, language, and memory deficits [34, 35] (see in Table 1). Though we classify these symptoms into categories, overlap is common. In a cross-sectional study comprising 310 patients with frontotemporal lobar degeneration (FTLD), 62% of participants met the diagnostic criteria for more than one syndrome [36]. Such high continuity in phenotypes challenges the current mode, which diagnoses patients as discrete syndromes [36].

**Table 1:** Phenotypes of tauopathies and their salient features

Phenotype		Salient features
*Cognitive/behavioral disorders*		
bvFTD	Behavioral variant of frontotemporal dementia	Disinhibition, apathy, loss of empathy, stereotyped behaviors, hyperorality
bvAD	Behavioral or dysexecutive variant AD	Milder behavior dysfunction compared with bvFTD and earlier memory loss
PSP-F	PSP with frontal presentation	Similar to bvFTD in initial stages
*Cognitive and movement overlap*		
PSP-RS	Richardson's syndrome	Postural instability, vertical supranuclear ophthalmoplegia, mild dementia
CBS	Corticobasal syndrome	Asymmetrical limb apraxia, parkinsonism, cortical function deficits
PSP-CBS	PSP-corticobasal syndrome	Similar to CBS
*Movement disorders*		
PSP-P	PSP with parkinsonism	Asymmetric onset of tremor, bradykinesia, rigidity
PSP-PGF	Progressive gait freezing	Start difficulty, freezing of gait
PSP-C	PSP with predominant cerebellar ataxia	Cerebellar ataxia
PLS	Primary lateral sclerosis	Upper motor neuron signs
*Language disorders*		
nfvPPA	Non-fluent/agrammatic variant of primary progressive aphasia	Laboured speech, sound errors, impaired grammar, Intact comprehension
svPPA	Semantic variant of primary progressive aphasia	Anomia, impaired comprehension, Intact grammar and comprehension
lvPPA	Logopenic variant of primary progressive aphasia	Anomia, fluency deficit, phonemic paraphasia and memory loss
PPAOS	Primary progressive apraxia of speech	Motor speech disorder
PSP-SL	PSP with predominant speech/language disorder	Similar to nfvPPA in initial stages
*Amnestic symptoms*		
aAD	Amnestic Alzheimer's disease	Prominent episodic memory loss and deficits in speaking, visuospatial disorientation, and impaired executive abilities

#### FTD

FTD is an overall depiction of three clinical variants, including behavioral-variant frontotemporal dementia (bvFTD), non-fluent variant primary progressive aphasia (nfvPPA), and semantic-variant primary progressive aphasia (svPPA), which predominantly affect personality, behaviors, and language skills [37].

Overlap of three phenotypes is more prominent as disease deteriorates, and is consistent with pathology expansion to the large areas of frontal and temporal lobes. Motor disorder can also appear over time [36].

bvFTD can be diagnosed with neuropsychiatric symptoms and behavioral changes [38]. Criteria include a) early disinhibition (inappropriate manner and irritability), b) apathy, which is similar to depression, c) loss of empathy, d) early compulsive, repetitive, or stereotyped behaviors, impaired executive abilities and e) eating pattern changes including varied preference and hyperorality [38–40]. nfvPPA shows labored speech, sound errors, and impaired grammar but still retains single-word comprehension and plain semantics. To the contrary, svPPA manifests anomia, impaired comprehension of single words and object knowledge, though grammar and speech fluency are spared.

#### PSPS

PSP syndromes (PSPS) encompass a range of behavioral, movement, and language disorders, among which PSP-RS (Richardson’s syndrome)—a classic movement disorder—is most widely studied. However, an autopsy based retrospective research indicated that up to 60–75% of patients with PSP pathology showed nonclassical variant PSP phenotypes which are outlined below, suggesting neglect in previous studies [41].

Postural instability and ensuing frequent falls are early signs of PSP-RS. But the diagnostic feature—vertical supranuclear ophthalmoplegia—usually develops in the 7^th^ year after onset, or never appears. Patients also show mild dementia, dysarthria, dysphagia, executive dysfunction and non-levodapa responsive parkinsonism (axial, symmetric akinetic-rigid syndrome, and extensor neck dystonia) [34, 42–44]. After the appearance of clinical symptoms, people tend to live for 6.9 years on average [43].

PSP with predominant parkinsonism (PSP-P) is considered the second most common phenotype in PSPS followed by PSP with corticobasal syndrome (PSP-CBS) according to a prospective study [45]. PSP-P can be easily misdiagnosed as PD in initial stages for their akin manifestations. Retrospective diagnosis is made when they develop PSP-RS related manifestations.

In patients diagnosed with PSP, the symptom of progressive gait freezing (PSP-PGF) occurs as early as the onset of disease and deteriorates during illness [44]. It’s a pure movement deficit with no response to levodopa and can happen under specific circumstances or is a feature of PSP-RS [44].

PSP and CBS are clinically and genetically overlapping, as 44% of patients with CBS had PSP-like features and 30% of patients with PSP had CBS-like features [36]. PSP-CBS patients have PSP pathology and similar symptoms to CBS, including levodopa-resistant rigidity, bradykinesia, and apraxia. Clinical differentiation between PSP-CBS and CBD-CBS is thus impossible. When compared with PSP-RS, PSP-CBS has more severe ideomotor apraxia [45].

PSP-speech language (PSP-SL) phenotype depicts a syndrome which initially develops language disorders akin to nfvPPA before transformation into PSP-RS after years. PSP with frontal presentation (PSP-F) shows similar symptoms to bvFTD in early stages before developing motor dysfunctions like PSP-RS. Cerebellar ataxia appears at the onset of PSP with predominant cerebellar ataxia (PSP-C), but is not included in MDS PSP criteria [46].

#### CBS

The corticobasal syndrome (CBS) is characterized by asymmetrical limb apraxia, levodopa-resistant parkinsonism (dystonia, rigidity, tremor, and myoclonus) and deficits in higher cortical function [41, 47, 48]. Initial symptoms usually include cognition decline, meaning problems in vision, language, executive ability, and social cognition [47]. Another typical feature for CBS is alien/anarchic limb phenomena [49]. Other common symptoms include dysarthria and dysphagia.

Akin to PSP-RS, CBS shows equally severe abnormalities in movement involving limbs and eyeball [45]. Nonetheless, the description of freezing gait and myoclonus is lacking in the definition of PSP-RS, which are still the vital characteristics of CBS and PSP-PGF [45].

#### CTE

Apart from typical history of head injuries, CTE (chronic traumatic encephalopathy) is reported to have nonspecific symptoms including emotional disorders (depression, anxiety, irritability), bradykinesia, gait instability and cognition impairment. However, bias is inevitable as most studies were conducted retrospectively. Recently, the first prospective study comparing the clinical profiles of 6 CTE and 25 AD patients with autopsy evidence didn’t find a distinct clinical phenotype from AD, showing similar performance in motor, neuropsychological, behavioral, and cognitive evaluations [50].

#### AD

Single or multidomain amnesia (also named aMCI, amnestic mild cognitive impairment) is the most common manifestation in early stages of typical AD (Alzheimer’s disease) [37, 51], in which patients’ ability to form new episodic memories is damaged while other functions are conserved [37]. Some symptoms might occur long time before diagnosis including depression, sleep pattern changes, apathy and severe anxiety [51]. Typical symptoms are more common in LOAD (late onset AD) patients.

In atypical AD, other than memory loss, abnormalities in language, visuospatial dysfunction, impaired behavior, and executive abilities tend to appear earlier, and mostly happen in EOAD (early onset AD) population [37, 51]. Based on clinical features, atypical AD has been classified into three phenotypes, including logopenic variant primary progressive aphasia (lvPPA), which has anomia, fluency deficit, phonemic paraphasias and memory loss; posterior cortical atrophy (PCA), which has compromised advanced visual functions (visual field cuts, alexia, agnosia, ideomotor apraxia and cortically blindness) in early stages; and behavioral dysexecutive variant AD (bvAD), which can be misdiagnosed as bvFTD but shows milder behavioral dysfunction and earlier memory loss [37].

### Pathology

#### 3R tauopathy

##### PiD

PiD is unique among tauopathies in predominantly obtaining 3R tau isoforms [1]. The atrophy of cortex is usually asymmetrical and presents as a knife-edge appearance, mostly occurring in frontal and temporal lobes, resulting in enlargement of the ventricle [52, 53]. The atrophy of neostriatum is inconsistent [52]. Other brain areas involved in PiD include amygdala and hippocampus [52] (see pathologies in Table 2).

**Table 2:** Neuropathological features of tauopathies

Neuropathology	Predominant tau isoform	Affected cell types	Affected brain areas	Pathology hallmarks
Pick’s disease	3R	Neurons and glia	Frontal lobes Temporal lobes Neostriatum Limbic system	Pick Bodies; Ballooned neurons/ Pick cells
Corticobasal degeneration	4R	Neurons and glia	Focal atrophy of superior frontal gyrus and parietal lobe	Pretangles; Astrocytic plaques (AP); Oligodendroglial coiled bodies (CB); Ballooned cells
Progressive supranuclear palsy	4R	Neurons and glia	Basal ganglia Brainstem Diencephalon Cortical regions	Globose neurofibrillary tangles (NFTs); Tufted astrocytes (TA); Oligodendroglial coiled bodies (CB); Neuropil threads
Globular glial tauopathy	4R	Neurons and glia	Frontal and temporal lobes Motor cortex Cerebral spinal tract	Globular oligodendrocytic incousions (GOIs); Globular astrocytic inclusions (GAIs); Neuronal cytoplasmic inclusions (NCIs)
Argyrophilic grain disease	4R	Neurons and glia	Anterior temporal lobe Amygdala Ambient gyrus Hippocampus	Argyrophilic grains; Coiled bodies; Neuronal pre-tangles; Ballooned neurons; Granular /fuzzy astrocytes
Aging-related tau astrogliopathy	4R	Astrocyte	Subpial, subependymal, perivascular, and white and grey matter areas	Thorn-shaped astrocytes (TSA); Granular-fuzzy astrocytes (GFA)
Chronic traumatic encephalopathy	3R + 4R	Neurons and glia	Hippocampus Neocortex Brainstem	Neurofibrillary tangles (NFTs); Astrocytic tangles
Primary age-related tauopathy	3R + 4R	Neurons	Temporal lobes Basal forebrain Brainstem Olfactory bulb	Neurofibrillary tangles (NFTs)
Alzheimer’s disease	3R + 4R	Neurons	Neocortex Limbic regions	Neurofibrillary tangles (NFTs); Amyloid plaques; Neuropil threads; Neuritic plaques

The orbicular, demarcated from cytoplasm, argyrophilic inclusions existing in neurons are typical features of PiD (named Pick bodies) [53]. Granule cells in the dentate gyrus of hippocampus are mostly affected [54]. Inclusions can also be found in pyramidal neurons and subcortical areas [52, 54]. Other pathology in PiD includes neuronal loss, gliosis, and ballooned neurons (or called Pick cells) [52, 54]. In glial cells, abnormal changes encompass thorn-shaped astrocytes and globular inclusions in oligodendrocytes [54].

#### 4R tauopathy

##### PSP

PSP mainly consists of 4R tau isoforms, with prominent neuronal tau pathology in subcortical areas and astroglial tau in neocortex and striatum [55]. Characteristic atrophy tends to occur in subthalamic nucleus and brainstem, particularly in midbrain tectum and the superior cerebellar peduncle [56].

Most tau pathologies have been found in neurons forming globular neurofibrillary tangles. In astrocytes, tau aggregates as tufts and surrounds nuclei in the proximal processes [57]. These special cells are called tufted astrocytes and are regarded as a feature of diagnostic value for PSP [56]. Other pathologic characteristics involve coiled bodies in oligodendrocytes, depigmentation in substantia nigra, gliosis and neuronal loss [56]. Herein, the severity of gliosis is associated with NFTs (neurofibrillary tangles) accumulations [58]. In some areas, like striatum (especially globus pallidus), grey matter in cortex and white matter in cerebellum, the presence of tau pathology in glial cells (astrocytes and oligodendrocytes) may precede tau accumulation in neurons, which might reflect the characteristic spreading pattern of different subtypes [55].

##### CBD

In CBD, more severe pathology is found in the frontal part of brain, while PSP mostly affects the hindbrain areas [59]. Frontoparietal cortices, striatum, amygdaloid body and the substantia nigra are also influenced in CBD [52, 60].

Both CBD and PSP have 4R tau inclusions in astrocytes, coiled bodies in oligodendrocytes, and dense neuropil threads, but their tau deposits in astrocytes have distinct morphology. Tufted astrocytes in PSP and astrocytic plaques found in CBD are mutually exclusive and haven’t been found in the same brain [61]. Similar to PSP, astrocytes in CBD might be involved earlier than neuronal tau accumulation [61] which is the diagnostic characteristic of CBD patients [55]. Astrocytic plaques and neuritic plaques are independent concepts with no overlap, although they sound akin and similar in appearance [52]. Considerable affected astrocytes exist in regional cortex and neostriatum [52] and have been proved to be associated with synapse loss [60]. In neurons, tau deposits are mainly composed of pretangles and reacts poorly to antibodies targeting NFTs [1, 52]. Ballooned neurons have also been found in CBD especially in limbic regions and cortex, and their density in CBD is 30 times higher than that in PSP [1, 52].

##### AGD

AGD is a 4R tauopathy characterized by circumscribed asymmetric atrophy in anterior temporal lobe, amygdala, and ambient gyrus [62].

Argyrophilic grains (AGs) are associated with cognitive decline, mostly seen in ambient gyrus initially and medial temporal lobe in later stages [63]. Granular/fuzzy astrocytes (GFAs) are constantly present in amygdala. Other brain regions variably affected include putamen, caudate nucleus and frontal lobe [64]. GFAs are astrocytes with slight perinuclear tau deposits and bushy tau granules gathering at astrocytic processes [64]. Moreover, the analysis of 105 cases hypothesized that AGD pathology is the strongest predictor of the presence of GFAs in amygdala [64]. Coiled bodies and neuronal pre-tangles can also be detected in AGD [1].

##### GGT

Tau pathology in globular glial tauopathy (GGT) mainly contains 4R tau isoforms, predominantly existing in oligodendrocytes and astrocytes [65]. Tau accumulations in neurons are distinguished by their diffuse distribution in the cytoplasm, where they form globular, cord-like, or horseshoe-shaped inclusions [52, 66].

The globular astrocytic inclusions (GAIs) in GGT, according to consensus, are distinct from those in other tauopathies, such as astrocytic plaques in CBD and tufted astrocytes in PSP, and all these astrocytic pathologies can appear in GGT [67, 68]. Furthermore, GGT is the umbrella term for three pathological subtypes [67]. In type I, globular oligodendrocytic inclusions (GOIs) are predominantly present in frontotemporal regions, leading to behavioral symptoms. Minor coiled bodies in oligodendrocytes as well as GAIs can also be found [67]. Movement is more heavily damaged in type II, where motor cortex and corticospinal tract are primarily affected [67]. Type III seems to be a combination of the two subtypes mentioned above, as frontotemporal lobes, motor cortex, anterior horn of the spinal cord and corticospinal tract are all involved [67–69]. Type III GGT has a higher burden and smaller size of GAIs in the motor cortex than type II. The horseshoe-like tau inclusions in neurons are exclusively present in type III GGT [66].

##### ARTAG

Aging-related tau astrogliopathy (ARTAG) mainly consists of 4R tau isoforms. It is characterized by thorn-shaped astrocytes (TSA) (mainly existing in subpial, subependymal, perivascular, and white matter areas) and granular-fuzzy astrocytes (often in gray matter) [70]. Chronic brain trauma might induce tau astrogliopathy, implying a connection between CTE and ARTAG [70]. Besides, ageing is proved to be the most important etiology of ARTAG [71].

#### 3R and 4R tauopathy

##### AD

AD obtains an equal amount of 3R and 4R tau in total and disproportional tau isoforms regionally [52]. NFTs and amyloid plaques remain as the typical characteristics, and tau-related pathology exists mostly inside neurons.

NFTs are mature forms of pretangles (mainly in somas and dendrites), comprising insoluble twisted fibers of mixed 3R and 4R tau. The intraneuronal flame-shaped NFTs can gradually become extracellular ghost tangles with reduced immunoreactivity [1].

Neuritic plaques, or senile plaques, are comprised of dystrophic neurites with tau pathology and extracellular Aβ deposits [72]. The Aβ deposition was found both in AD and cerebral amyloid angiopathy (CAA), suggesting a cross-talk between them [73]. Moreover, the research on relationship between neuritic plaques and cerebrovascular dysfunction have long focused on Aβ deposits [73]. The less discussed tau neurites were linked to compromise of the microvasculature in a recent study [72].

Furthermore, Aβ might accelerate tau deposits as more tau PET (positron emission tomography) tracers (18F-AV-1451) have been taken up by neurons with more amyloid accumulations [74]. A 7-year prospective study discovered a sequential occurrence of increase in Aβ, tau accumulation and cognitive decline, and the initial Aβ level was related to successive tau fluctuations and ultimate tau concentration [75]. Another study further indicated that the ratio of Aβ42/40, rather than total Aβ levels, plays a key role in the induction of NFTs [76].

##### CTE

Chronic traumatic encephalopathy (CTE) is a progressive tauopathy comprising both 3R and 4R tau isoforms. While a mixed expression of 3R and 4R tau is found in neurons, astrocytes only contain 4R tau [77]. In addition, the chief composition of tau aggregates in CTE is hypothesized to experience a transition from 4R tau (I-II stages) to 3R tau (III-IV stages) in company with exacerbation of tauopathy and growing amount of extracellular tau (ghost tangles) [78].

Typical pathology of hyperphosphorylated tau in neurons is primarily found in the neocortex (especially dorsolateral frontal cortex and superior temporal cortex), entorhinal cortex, amygdala, locus coeruleus, and hippocampus (especially subareas CA4 and CA2/3) [78, 79], which is associated with disease severity and aids in the differentiation from AD. Similar pathology also shows preference for perivascular regions deep in the cortical sulcus [77, 78].

##### PART

Primary age-related tauopathy (PART) describes pathology that consists of AD-like NFTs and few or no Aβ plaques [80]. In contrast to AD, NFTs in PART are mainly circumscribed in temporal lobes, basal forebrain, brainstem and olfactory bulb [80]. Whether PART is a distinct pathology or represent early stages of AD is under discussion [81].

In addition to morphological variations, a growing body of literature suggests a direct correlation between distinct tau fibril structures and neuropathological patterns, supporting the concept of tau strains (pathological conformations that linked to defined neuropathological patterns) [82]. Recently, a study proposed a classification based on disease-specific tau filament folds, providing a novel perspective complementary to clinical symptoms and autopsy study [83]. Further exploration of unique conformations of tau will assist in the selection of specific tau tracers and antibodies [82].

### Progression of tau pathology

As disease deteriorates, tau pathology is hypothesized to sequentially invade wider areas of the brain, which is observed in animal models, autopsy samples (using immunohistochemical staining) and living people (using tau-PET). The propagation of tau is described as “prion-like”, due to its seeding (transforming normal tau into misfolded protein) and transmission properties, though lacking evidence of interpersonal infection. A growing body of literature suggests heterogeneity in propagation routes within one pathology, probably reflecting different phenotypes, and might imply differences in neuropathology and ensuing therapies. In this section, we briefly summarize generally accepted propagation patterns and their recent progress.

#### PiD

**Fig. 2:**
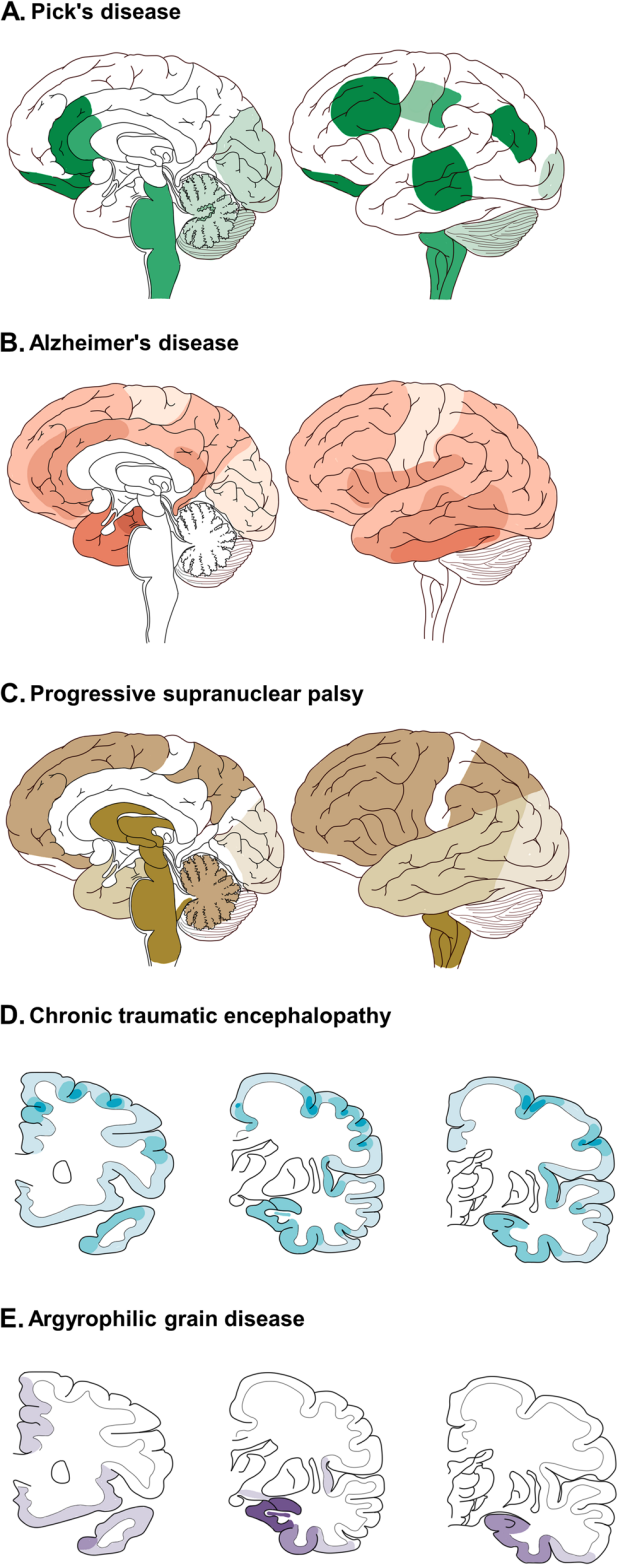
Spatial–temporal distribution of tau lesions in schemes of brains. Deeper color means earlier involvement. **A** Tau progression in PiD has four stages, shown in medial and lateral views. Stage I, tau affects angular gyrus, limbic and frontotemporal regions. Stage II/III involves white matter tracts, subcortical structures (thalamus, striatum), serotonergic/noradrenergic brainstem nuclei, primary motor cortex and pre-cerebellar nuclei. Stage IV, tau invades visual cortex and cerebellar. Modified from [84]. **B** Tau progression in AD has six stages. Stage I/II, tau affect transentorhinal area. Stage III/IV, severe involvement of entorhinal, hippocampus and limbic areas happens. Stage V/VI, tau reaches primary and secondary neocortex. Modified from [85, 86]. **C** Tau progression in PSP has seven stages. Stage 0/I, pallido-luyso-nigral axis shows tau burden. Stage II/III, tau invades basal ganglia, dentate nucleus and pedunculopontine nucleus. Stage IV/V, tau reaches frontoparietal and temporal lobes. Stage VI/VII, occipital cortices, substantia nigra, subthalamic nucleus and globus pallidus are involved. Modified from [55]. **D** Tau progression in CTE has four stages, shown in coronal view at levels of genu of corpus callosum, mammillary body, and lateral geniculate body. Stage I/II, tau is restricted focally deep in the sulci of cortex, especially frontal lobe, surrounding small vessels and expand to superficial layers. Stage III, tau is widespread to cortices including frontal, temporal, parietal, and insular lobes. Meanwhile, amygdala, hippocampus, and entorhinal cortex are involved. Stage IV, tau affects most regions of cerebral cortex. Modified from [87]. **E** Tau progression (argyrophilic grains (AGs)) in AGD has four stages, shown in view same as D. Stage I is characterized by AGs in ambient gyrus, hippocampus (CA1), entorhinal and amygdala. Stage II shows involvement of medial temporal lobe and subiculum. Stage III, AGs reaches anterior temporal, cingulate gyrus, rectus gyrus, septum, accumbens nucleus, insular and orbitofrontal cortices, and hypothalamus (CA2 CA3). Stage IV involves neocortex and brainstem (not shown in figures). Modified from [63]

A four-stage sequential pattern for PiD pathology was proposed in 2016 based on neuropathological examination [84]. Tau deposits usually start from frontotemporal neocortex and limbic areas and move to subcortical regions, brainstem, motor cortex and finally the visual cortex [84] (Fig. 2A).

In early stages, discrepancies in areas of involvement exist between the bvFTD-PiD and the nfvPPA-PiD populations. bvFTD-PiD has tau pathology predominantly in prefrontal and temporal cortices, while nfvPPA-PiD mainly affects left inferior frontal, orbitofrontal and insula cortices [88]. Later, pathology in both groups spreads to motor areas and parietal lobes [88].

#### AD

A stereotype about the propagation of NFTs has been proposed as the six Braak stages, which ignores the individual variabilities. To improve the prediction of tau spread, a model was proposed based on tau-PET samples and hypotheses about tau spreading along neuronal connections (Fig. 2B) [85].

Moreover, adopting tau-PET tracer instead of histopathological staining, heterogeneity in spreading patterns was suggested by a study, comprising limbic-predominant phenotype, parietal-dominant and medial temporal lobe-sparing phenotype, predominant posterior occipitotemporal phenotype, and asymmetric temporoparietal phenotype, which might unify the propagation patterns of typical and atypical AD [89].

#### CTE

The tau pathology in CTE starts from neocortex and spreads to medial temporal lobe [78]. Gradually, neuronal and glial pathology will expand to extensive neocortical, brainstem and allocortical areas [77]. In 2013, based on the severity and distribution of hyperphosphorylated tau, McKee and colleagues divided the progression of pathology into four stages [87] (Fig. 2D). In a recent study, McKee staging scheme is consistent with higher scores of p-tau density, older age, and propensity for dementia, which proved its value in CTE research [79].

#### PSP

A recent study hypothesized that all PSP subtypes share one common site for the initial development of neuronal pathology, which is the pallido-nigroluysian axis [55]. Apart from neuronal tau, discrepancies in cell-specific tau distribution (especially tau deposits in astrocytes and oligodendrocytes) exist among different phenotypes, suggesting varying propagation routes and dynamics [55]. Analysis based on pooled cases ignoring clinical phenotypes suggests tau progression from subcortical areas to cortices [55] (Fig. 2C).

#### AGD

A four-stage theory depicting the chronological progression of AGs was proposed. Those brain areas include ambient gyrus as the initial site, temporal lobe, subiculum, entorhinal cortex, septum, insular cortex, and anterior cingulate gyrus [57] (Fig. 2E). In wildtype mouse models, after inoculating pure AGD pathology unilaterally in hippocampus, involvement of fimbria, bilateral corpus callosum and wider brain regions was observed, proving the seeding and spreading property of AGD tau [90].

#### GGT

Tau propagation in GGT type I is somewhat described, invading white matters in peri-amygdala and hippocampal regions initially, and expanding to limbic regions [57]. Reports about tau progression in GGT type II and III haven’t been found.

### Clinicopathological correlations

**Fig. 3:**
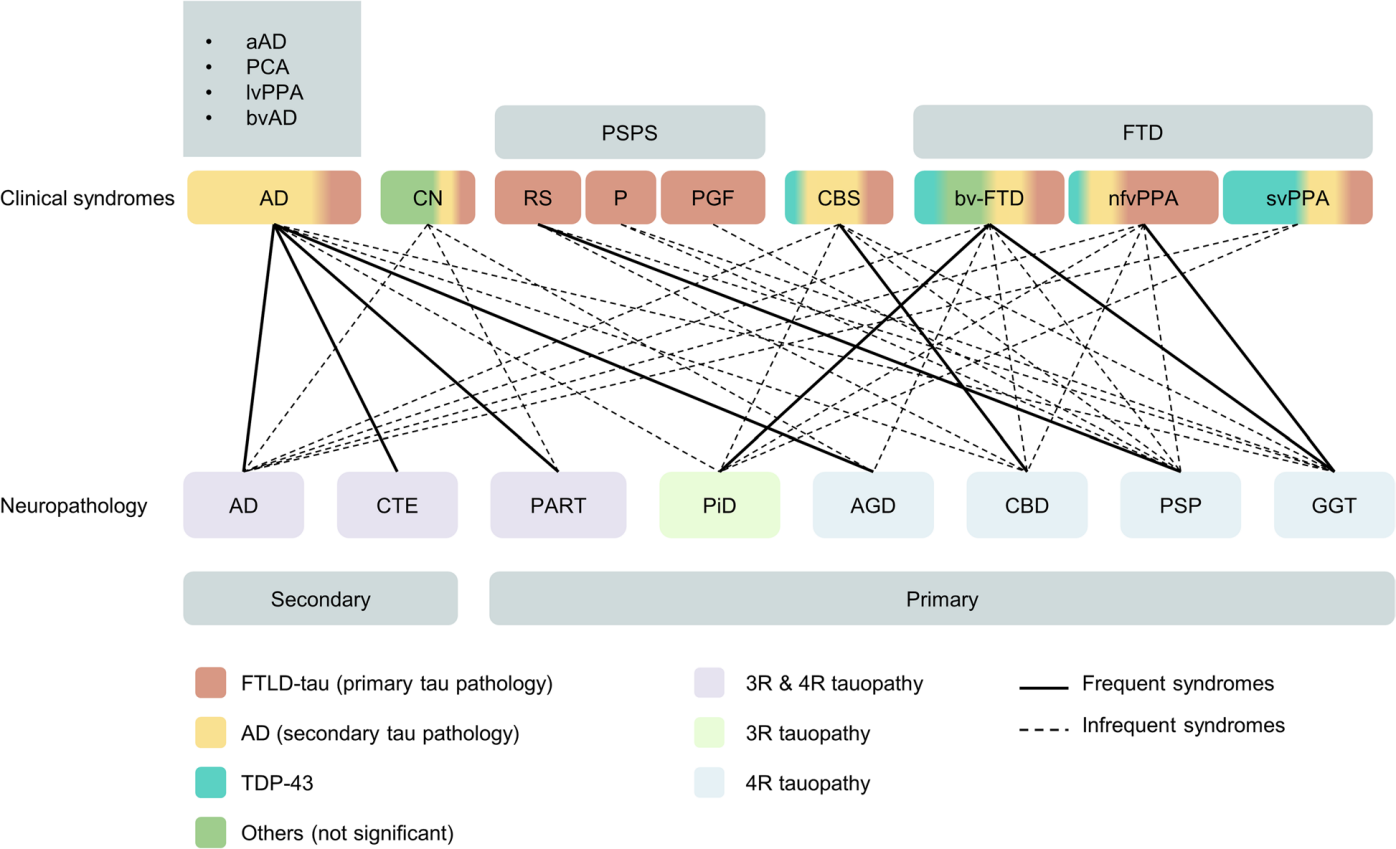
Clinicopathological correlations of tauopathies. In the row listing clinical syndromes, combination of colors depicts the pathology composition within one phenotype. In the row listing neuropathology, different colors represent the classification of tauopathies based on tau isoforms primarily exist in aggregates. The solid lines connect pathologies and their frequently associated phenotypes, while dotted lines show infrequent associations. *AD* Alzheimer’s disease, *aAD* amnestic AD, *PCA* posterior cortical atrophy, *lvPPA* logopenic variant primary progressive aphasia, *bvAD* behavioral dysexecutive variant AD, *CN* cognitively normal, *RS* Richardson syndrome, *P* parkinsonism, *PGF* progressive gait freezing, *CBS* corticobasal syndrome, *FTD* frontotemporal dementia, *bvFTD* behaviour variant of frontotemporal dementia, *nfvPPA* non-fluent/agrammatic variant of primary progressive aphasia, *svPPA* Semantic variant of primary progressive aphasia, *CTE* chronic traumatic encephalopathy, *PART* primary age-related tauopathy, *PiD* Pick’s disease, *AGD* argyrophilic grain disease, *CBD* corticobasal degeneration, *PSP* progressive supranuclear palsy, *GGT* globular glial tauopathy

The clinical symptoms of tauopathies mainly imply brain regions developing pathology. However, anatomical and pathological overlap among tauopathies, as well as participation of other proteins, disrupts the consistent one-to-one match between clinical symptoms and underlying pathology. For example, in AD patients with pathology evidence, most of them show symptoms of AD, but they can also present as CBS, bv-FTD and nfv-PPA. In clinically diagnosed AD patients, apart from AD pathology, CTE, PART, PiD and AGD can also appear (Fig. 3).

#### 3R tauopathy

##### PiD

PiD shows symptoms of FTD, with bvFTD in the beginning and gradual impairment in social comportment and executive skills [84]. In a retrospective study analyzing 21 patients with Pick’s disease as pathology, more than half of them showed symptoms like bvFTD (12/21), making it the most common one [53]. Less common manifestations include PPA (primary progressive aphasia) (7/21), CBS (1/21) and amnestic dementia (1/21) [53]. These presentations are in line with the brain areas that tend to be affected in PiD [52]. Besides, PiD patients presenting as bvFTD has faster rate in frontotemporal atrophy when compared with those having PPA manifestations [88].

#### 4R tauopathy

##### AGD

AGD is associated with ageing, lower income and eating disorders [27]. In a population aged 80 years or older, people with AGD pathology constitute more than half of it [52]. Even in a population with normal cognition, 31% of them might bear insidious AGD pathology, eliciting dispute about the pathogenetic role of AGD [27]. Coexistence with other pathologies is common. Overlaps with PSP, CBD, AD, PiD, PD, LBD (Lewy body dementia), etc. have been found [27, 52].

Clinicopathological correlations are not specific, which is confounded by normal ageing and concurrence of pathologies, resulting in difficulties of reaching antemortem diagnosis [52]. A retrospective study on 55 patients aged under 75 with relatively pure AGD pathology (low concurrence) concluded common symptoms for AGD patients: progressive cognitive impairment akin to the onset stage of AD, urinary incontinence, seizures, and psychological disorders [28].

##### CBD

Only 25–50% of CBD patients manifest as CBS. Apart from that, bvFTD, nfvPPA, AD, and Richardson’s syndrome also appear in CBD cases [61]. In patients with rapid progression (RP-CBD, disease duration is less than 3 years), rapid global cognitive impairment, CBS and Richardson’s syndrome are more common and are accompanied by advanced pathology (predominant neuronal tau deposits and nigral cell loss) [91]. By analyzing 210 cases from brain banks, CBS occupies 37.1% of CBD cases, followed by Richardson’s syndrome (23.3%), bvFTD (13.8%), AD-like dementia (8.1%) and nfvPPA (4.8%) [48].

A definite diagnosis needs specific biomarkers and genetic information. Thus, current clinical diagnostic criteria are relatively broad to obtain more possible cases [48].

##### PSP

The Movement Disorders Society PSP diagnostic criteria published in 2017 lists syndromes that possibly imply PSP pathology, which have already been mentioned in the previous part [92]. 24–50% of PSP patients manifest as PSP-RS, followed by PSP-P [57]. Primary lateral sclerosis showing upper neuron disorders is reported in rare cases [57].

Overlap with other pathology is common (especially with AD and AGD) but has little impact on disease progression [93]. Pathology also varies among different phenotypes. In a prospective study, PSP-CBS had the lowest score in semantic fluency test, followed by PSP-RS, implying the most severe damage to left inferior frontal and temporal cortex in both phenotypes [45]. Studies have found more involved glial cells in PSP-RS when compared with PSP-P. In the meanwhile, PSP-P exhibits more benign clinical progression [55]. A heavier burden of total tau in frontal and temporal neocortical neurons and white matter is more common in PSP-FTD when compared with PSP-RS [94].

##### GGT

According to the information from 88 GGT patients worldwide, the most common phenotypes for GGT are PPA (25%) and bvFTD (22.7%), less common ones include upper motor neuron disorders, amnesia, Richardson syndrome, parkinsonism and CBS, similar to phenotype composition in GGT with MAPT mutations [95].

Three GGT pathology subtypes are associated with varied phenotype spectrums which is consistent with typical affected brain regions, but is not enough for neuropathological differentiation [95]. Type I GGT predominantly presents as bvFTD due to damage in frontal and temporal lobes. Type II is characterized by upper neuron symptoms, including Richardson’s syndrome eliciting discussion about whether type II GGT belongs to atypical PSPs. Type III presents as a combination of type I and type II, involving bvFTD, movement disorders and extrapyramidal syndromes [95].

#### 3R and 4R tauopathy

##### AD

Patients with AD pathology can manifest as heterogenous phenotypes, probably caused by comorbidity and regional vulnerability [96]. EOAD has more concomitant pathologies, which aligns with a lower cognitive score before death [97]. Furthermore, the presence of LBD is frequently associated with a faster decline in cognition [96]. Typical AD has damage primarily in limbic system and the medial temporal lobe, while PCA locates damage in occipitoparietal region and, in rare cases, the posterior temporal lobes [37].

NFT is a hallmark closely associated with AD’s manifestations. It is a better predictor of the severity of symptoms (especially cognition decline) and neuronal loss than amyloid beta peptide [3, 98]. Intriguingly, patterns of brain atrophy implied by tau deposits can precede and be identical to later detection by MRI and FDG-PET [99, 100]. Meanwhile, different AD phenotypes show higher NFT burden in specific brain regions. The superior temporal gyrus has more severe NFT pathology in lvPPA; in CBS, higher NFT burden is found in primary motor cortex; in visuospatial dysfunction, NFT tend to accumulate in angular gyrus and hippocampus (CA1 region) [96].

##### CTE

Clinical symptoms of CTE might largely depend on concomitant pathology [50]. Thus, CTE’s contribution to clinical manifestation is uncertain. It’s mostly accompanying other pathologies, or in fewer cases, the primary neuropathological change driving symptoms, or without clinical significance [101]. In a retrospective study, athletes with CTE pathology mainly received AD and vascular dementia as their clinical diagnosis [101].

The exacerbation of symptoms is associated with the progression of tau pathology. In stage I CTE, headache and distraction are most common. Amnesia and mood disorders happen in stage II. Impairment of cognition, executive function and visuospatial ability appear in stage III. At last, stage IV patients show dementia with short-term memory loss and mood disorders [87]

### Genetics

What provokes the conversion from soluble disordered tau to insoluble misfolded deposits is still unknown, so does the mechanism by which tau leads to neurodegeneration. Studies in genetics offer a way to discover and understand potential pathophysiological processes.

#### MAPT mutations

In primary tauopathies, sporadic cases constitute the majority of the incidence. Only approximately 31% have been reported with family history [102], which is even lower in PSP and GGT [69]. Familial inheritance is usually in an autosomal dominant pattern in FTD and is associated with three different genes (C9orf72, GRN, or MAPT), of which MAPT occupies 5–10% [21, 102]. While in secondary tauopathies, including AD, myotonic dystrophy, Down syndrome, etc., no pathogenic MAPT mutation has been found.

Currently, over 50 mutations in the MAPT gene have been correlated with neurodegenerative diseases [2]. In rTg4510 mice, overexpression of tauP301L leads to NFTs accumulation, memory disorders, rapid neuronal loss, and brain atrophy in early stages [103]. Possessing more than one mutated site on the MAPT gene might have additive effects, as expressing tau with two mutations (P301L and S320F) in CNS led to severer tangle pathology, neuroinflammation and memory deficits than tau with a single mutation [104]. Some pathogenic MAPT mutations are shown in Fig. 1. Missense mutations are most common, followed by silent or nonsense mutations and duplicates or deletions.

In sporadic and familial PSP, MAPT is the strongest genetic risk factor. More than 15 mutations have been identified [105]. In GGT, MAPT mutations include N296H, R5H, K317M, K317N and P301L, mainly distributing in exon 10 and its flank [69]. In the CBS population, MAPT is the second most common risk gene, and mutations are all missense variants [106].

A growing body of literature elucidates the downstream events of tau mutations, including enhanced tau aggregation, dysfunction in mRNA splicing and tau structure alteration [7].

MAPT mutations usually happen in the carboxy-terminus where microtubule binding domains (MTBDs) exist. Thus, the binding affinity of tau is often impaired, resulting in microtubule destabilization and tau aggregation [102]. Unlike most MAPT mutations, Q336H and Q336R in exon12 enhanced MT-tau binding and reduced phosphorylation of tau, but still led to increased seeding propensity, tau aggregation and risk for PiD [7, 107]. In this condition, hyper-stabilization of MT and other molecular mechanisms might contribute to pathology [7, 107].

Besides, multiple exonic and intronic variants within and around exon 10 can impact splicing of pre-mRNA (E10 exclusion: ΔK280, G272V; E10 inclusion: N279, L284L, S285R and c.823-10G > T) [108]. Consequent unequal ratio of 3R and 4R tau isoforms might generate more free-floating tau monomers, which will enhance tau aggregation [3, 4, 7].

In regions far from MTBD, the role of mutations is not clearly elucidated. A shift of alanine to threonine at codon 152 in MAPT might confer a new phosphorylation site, leading to impaired MT binding, disordered retrograde axon transport and an increased risk for primary and secondary tauopathies [109].

#### MAPT polymorphisms and haplotypes

The largest linkage disequilibrium (LD) block on chromosome 17 contains MAPT gene and comprises two haplotypes (H1 and H2) [110, 111]. 3 million years ago, the H2 haplotype was generated through a 900 kb inversion from H1 and only appears in the Caucasian population [110–112].

In CBD and PSP, the H1 haplotype is overexpressed and has been verified as the strongest genetic risk factor [59, 108, 112]. Meanwhile, specific sub-haplotypes of H1 that contribute to tauopathies need further elucidation [111].

A case–control study involving 802 autopsy verified PSP patients has discovered that H1d (OR: 1.86), H1g (OR: 3.64) and H1o (OR: 2.60) could lead to the onset of PSP [111], and H2, H1c, H1e, H1q and H1d are related to the severity of tau pathology [111].

As PSP and CBD share overlapped genetic risk background [59], similar haplotypes including H1d (OR: 1.76) and H1c (OR: 1.49) also appear to be risk factors for CBD, but they can’t predict or influence the severity of pathology or course of disease [59]. In contrast, H2 is associated with a lower risk of disease (OR: 0.26) [59].

The association between MAPT haplotypes and AD is inconsistent in previous researches [110]. Recent studies have described the H2 haplotype as playing a protecting role in the occurrence of LOAD (OR = 0.90) [113]. A stratification analysis comprising 4124 AD and 3290 healthy controls concluded that the H1 haplotype mildly increased risk for AD only in people without APOE ɛ4 [114], as the contribution of the H1 haplotype to disease risk might be covered up by APOE ɛ4 in its carriers.

Increased expression of MAPT, especially 4R tau isoforms, is associated with the H1 haplotype, being 1.5 times more than that in the H2 haplotype [113]. Such amount of tau accelerates the toxicity of Aβ and formation of tau oligomers in drosophila, leading to an increased propensity for LOAD [113]. While the H2 haplotype, exhibiting protective effect in most cases, can reduce the alternative splicing of E10, generating more 3R tau isoforms [2].

#### Risk loci beyond MAPT

To date, GWAS (Genome Wide Association Study) studies have identified more than 10 genes contributing to the risk of PSP. LRRK2 is a relatively rare cause, and is highly associated with PD [105]. A SNP (single nucleotide polymorphism) rs2242367 in LRRK2 was recently proved to be highly correlated with survival of PSP patients [43]. The mutation upregulated lncRNA (LINC02555) and influenced the expression of LRRK2 [43]. The relationship between MOBP, STX6 and PSP, proposed by the largest GWAS of PSP to date [115], was confirmed again recently in a European population [116]. By analyzing associations between PSP latent traits and risk SNPs, a study identified 16 new candidate genes (SPTBN5, EHD4, SEC13, ATP2B2, etc.) that potentially correlated with PSP pathology [117].

In addition, the intronic variant rs564309 in TRIM11 is more common in non-RS phenotypes [105]. TRIM11 helps to degrade misfolded proteins via UPS (ubiquitin proteasomal system) [105] and variant rs564309 can thus lead to increased NFTs but doesn’t influence glial pathology [118].

In a systematic review of 58 reported CBS cases, GRN was the most common genetic risk factor, followed by C9ORF72, PRNP, GBA and MRS2/ZHX2 [106]. CBD and PSP have many risk genes in common, including MOBP, CXCR4, EGFR, GLDC and VEGF. A combination of MAPT and CXCR4 increases the risk of PSP, while the presence of MAPT, MOBP, and GLDC increases the susceptibility to CBD or FTD [106]. In the first research exploring associations between mitochondrial DNA (mtDNA) and susceptibility to PSP and CBD, mtDNA sub-haplotype H4 contributed to an increased risk of CBD, and haplotype HV/HV0a can be a marker for PSP pathology as it corresponded with decreased neuropil threads [119].

APOE 4, APP, PSEN1, and PSEN2 are well-known in Alzheimer's disease. Apart from that, a recent meta-analysis identified new causal genes, including CCDC6, TSPAN14, NCK2, SPRED2, BIN1, APH1B, PTK2B, PILRA, and CASS4 [120]. In a genetic meta-analysis, risk loci for LOAD concerning tau protein and APP metabolism were confirmed, showing shared genetic consequences as EOAD with autosomal dominant variant [121]. These new risk genes were IQCK, ACE, ADAM10, ADAMTS1, and WWOX, and they also affected immunity and lipid metabolism in this study [121].

APOE ɛ4 is a well-established risk factor for AD and decreases survival in a dose-dependent manner, while APOE ɛ2 significantly reduces the risk of developing AD [122]. The relationship between APOE and atypical parkinsonism is controversial. Previous research regarded APOE ɛ2 as a risk factor for PSP, AGD [57]. However, a recent study hasn’t found significant associations between APOE and the risk of PSP or CBD [122].

Further work focusing on functions of risk loci is important as most of them locate in noncoding regions [123, 124]. Recently, the employment of machine learning in integrating the multi-omic framework offered an approach to predict noncoding variants’ functions [123]. In another research, 27 top risk loci for AD altered the expression of 21 adjacent genes. Seven SNPs (particularly in INPP5D, PTK2B) were linked to tau aggregation, 11 SNPs (in ADAM10, IGHV1-68, SLC24A4/RIN3, etc.) were linked to amyloid pathology and 8 SNPs (especially in ECHDC3, HS3ST1) were linked to neurodegeneration [124].

### Tau biomarkers

In recent years, advancement has been made concerning in vivo detection of tau, which mainly consist of neuroimaging, cerebrospinal fluid, and blood markers. They are auspicious candidates for early and differential diagnosis, disease progression prediction, pharmacodynamic reflections, therapy mechanism interpretation [125] and trials participants selection [126].

At the same time, autopsy studies are also needed to validate sensitivity and specificity of biomarkers and detect concomitant pathology. Moreover, various tau related biomarkers have been explored more thoroughly in Alzheimer's disease [127], while many non-AD tauopathies still don’t have commonly accepted tau related biomarkers [128, 129].

#### CSF and blood tau

##### CSF biomarker

CSF t-tau can reflect neuronal injury and is common in various neurodegenerative diseases. CSF p-tau has stronger correlations with pathologic state [130] and CSF p-tau181 (tau phosphorylated at threonine 181) is exceedingly specific for AD, which facilitates differential diagnosis.

However, during the progression of AD, increases in CSF p-tau217 (tau phosphorylated at threonine 217) are significantly higher than CSF p-tau181, suggesting a better performance of CSF p-tau217 in the diagnosis of early and advanced stages of AD [131]. Another study found a stronger association between CSF p-tau217 and PET imaging of tau and amyloid. Since amyloid is specific for AD, p-tau217 allows for a more accurate distinction between AD and non-AD tauopathies (like FTLD) [132]. In addition, CSF p-tau181 and p-tau217 show subtle differences when acting as prognostic markers predicting cortical thickness [133].

Other phosphorylated tau proteins are also promising biomarkers. CSF p-tau235 increases early during the course of AD, and the presence of CSF p-tau235 and CSF p-tau231 follows sequential phosphorylation incidence, which can be used in the staging of early phases [134]. However, CSF t-tau and p-tau decreased in PSP patients compared with healthy control, and lower baseline p-tau was associated with more rapid progression of disease, suggesting a different tau pathogenesis from AD [135].

Tau 368 is a tau protein fragment ending in amino acid 368. It increases in the brain tissues of AD patients, constituting tangles. Thus, the ratio of CSF tau 368/t-tau decreased in all AD cohorts in a study, reflecting tangle pathology [136].

As an axonal protein, CSF neurofilament light (NfL) chain is a non-specific biomarker for neurodegeneration. It is associated with cingulum and corpus callosum FA (fraction anisotropy) and WMH (white matter hyperintensities) in MRI [137]. In AD patients, CSF NfL correctly identified more AD cases than CSF t-tau [138]. In FTLD patients with autopsy evidence, CSF NfL increased and showed higher accuracy than t-tau in differentiating FTLD pathology from normal participants and AD, even in the context of co-pathology [138].

The development of 4R-tau-specific CSF biomarkers is currently underway. By adapting the ultrasensitive tau seed amplification assay (4R RT-QuIC), a study analyzed CSF from living people and postmortem samples diagnosed as PSP and CBD, and identified three 4R tau seeds associated with diseases [139], which needs further validation. Such method was also applied to detect 3R and 3R/4R tau aggregates in AD, CTE and PiD [140].

CSF biomarkers have limitations such as high cost, limited availability, and invasive quality [126, 141]. Hence, research on blood-based biomarkers covering p-tau18, p-tau217, plasma NfL, etc. provides a rosy future [142].

### Plasma biomarker

As a biomarker specific for AD, plasma p-tau181 can discriminate AD from healthy controls and FTLD efficiently [143, 144]. Although it can’t predict disease progression alone [145], higher p-tau181 is associated with the conversion of MCI to AD and cognitive decline [143].

Plasma p-tau217 helps to differentiate AD from other neuropathological diseases (like FTLD), with a higher accuracy than plasma p-tau181 [146], plasma NfL, and MRI measures and shows almost equal efficacy compared with CSF p-tau and tau-PET [142]. Higher baseline p-tau217 levels can predict disease progression independently (exacerbation in brain atrophy, tau deposits and cognition decline), even outperforms CSF biomarkers [145]. But p-tau217 failed to act as a prognostic tool in non-AD tauopathies [145]. Furthermore, the ability to distinguish between normal and abnormal tau PET imaging made p-tau217 a possible replacement for tau PET when it didn't take into account regional deposits [142, 146].

P-tau biomarkers are promising tools for preclinical diagnosis as phosphorylation precedes aggregation. A study found an increase of CSF and plasma p-tau181, CSF p-tau217 and CSF p-tau231 in pre-symptomatic stages of AD, which is useful in further clinical trials on early-stage AD patients [147]. Another study found plasma p-tau217 increases before the tau-PET signal [125], with performance in early diagnosis close to CSF p-tau [142]. Moreover, a combination of plasma p-tau, APOE genotyping and cognitive test can accurately predict AD diagnosis in the subjective cognition decline and MCI population [148].

The level of NfL in plasma is highly associated with CSF NfL and varies rapidly around the onset of symptoms in AD patients [149]. It can imply neuronal damage [126] and monitor neurodegeneration and disease progression [149]. In addition, NfL is a potential biomarker for non-AD tauopathies. Plasma NfL increases in FTLD and can differentiate FTLD from healthy controls and AD patients, with better performance than plasma t-tau [150]. Another study found NfL in serum along with cognitive screening could differentiate PD from PSP and CBS [41].

Despite the advancement, the accuracy of biomarkers in plasma might be affected by dilution, peripheral alterations including degradation by liver and kidney, and disturbed by peripheral analogous proteins when compared with evaluation in CSF [126, 151].

#### PET

In 2020, a first-generation tau tracer [^18^F]-flortaucipir (AV1451) was authorized as assistance for evaluations of NFTs in  AD by the US Food and Drug Administration [2]. Recently, the significant correlation between ^18^F-AV1451 and NFTs distribution has been proved again in a study comparing tau PET imaging with postmortem assessment (sensitivity: 94.7%, specificity: 50.0% [152]). ^18^F-AV1451 can reflect advanced tau pathology in AD and is more relevant to dynamic clinical changes [153], but it failed to detect early pathology formation as CSF p-tau does [154], and didn’t reliably predict tau pathology in non-AD patients [154], suggesting its use mainly in the neuropathological diagnosis of AD [155]. In preclinical stages of AD, BP_ND_ (binding potential) of ^18^F-AV1451 subtly elevated in MAPT mutation carriers (especially R406W) compared to controls, suggesting its use as an early prediction for MAPT mutations that lead to 3R/4R tauopathies [156]. Similar phenomenon was observed in FTD patients with Q351R MAPT mutation [157]. ^18^F-AV1451 uptake can more accurately predict cognition decline and neurodegeneration in AD than CSF p-tau [153], while another study found CSF p-tau elevation preceding 18F-AV1451 positivity during cognition decline [155]. Moreover, researchers have found uptake of this tracer in FTLD with TDP-43, raising the controversy about the specificity of ^18^F-AV1451 for tau pathology, especially when used in FTLD patients [158].

[^18^F]-JNJ-067 is also exceedingly selective for AD and barely binds tau protein in healthy controls, MCIs and PSPs [159]. This quality offers a chance to use [18F]-JNJ-067 in differentiating AD from healthy seniors and other tauopathies [159]. Its use in the early clinical phase is limited due to insufficient sensitivity to differentiate healthy controls and MCIs [159].

[^18^F]RO948 and [^18^F]MK6240 perform better in AD patients. A study only found retention of [^18^F]RO948 in AD cases and R406W mutation carriers when compared with MCI and other neuropathological diseases, suggesting use for differentiation [160]. Intriguingly, for ^18^F-AV1451, [^18^F]RO948 and [^18^F]MK6240, tracer uptake in the temporal lobe (with similar cut-offs around 1.35 SUVR) is a common feature for differentiating AD from healthy seniors and non-AD tauopathies [161]. What’s more, [^18^F]MK6240 has got a higher dynamic range than ^18^F-AV1451, which is promising in detecting earlier pathologies [162].

In non-AD tauopathies, tau tracers usually show an absent-to-low binding level, and development is underway. [^18^F]PM-PBB3, derived from ^11^C-PBB3, was more stable and thus elevated the sensitivity of detecting deposits, especially in FTLD tauopathies, which possess fewer fibrillary aggregates than AD [163]. Compared with 3R/4R tauopathies, [^18^F]PI-2620 is less stable when binding to 4R tau in CBS and PSP, but shows different uptake patterns in cortices, supporting its role in differentiation [164]. [^3^H]CBD-2115 shows higher affinity for 4R tau than [^18^F]MK6240 and ^18^F-AV1451 in PSP and CBD, and further improvement will focus on BBB (blood–brain barrier) penetration and binding affinity [165].

At present, off-target is still a problem existing in all tau tracers to varying degrees. The elevation of specific binding is useful in accurate quantification. Multiple qualities are suggested for ideal tracers: higher dynamic range, lower off-target incidence, steadiness, and higher consistent reliability [159].

### Targeting tau therapeutics to tauopathies

#### Microtubule stabilization

**Fig. 4:**
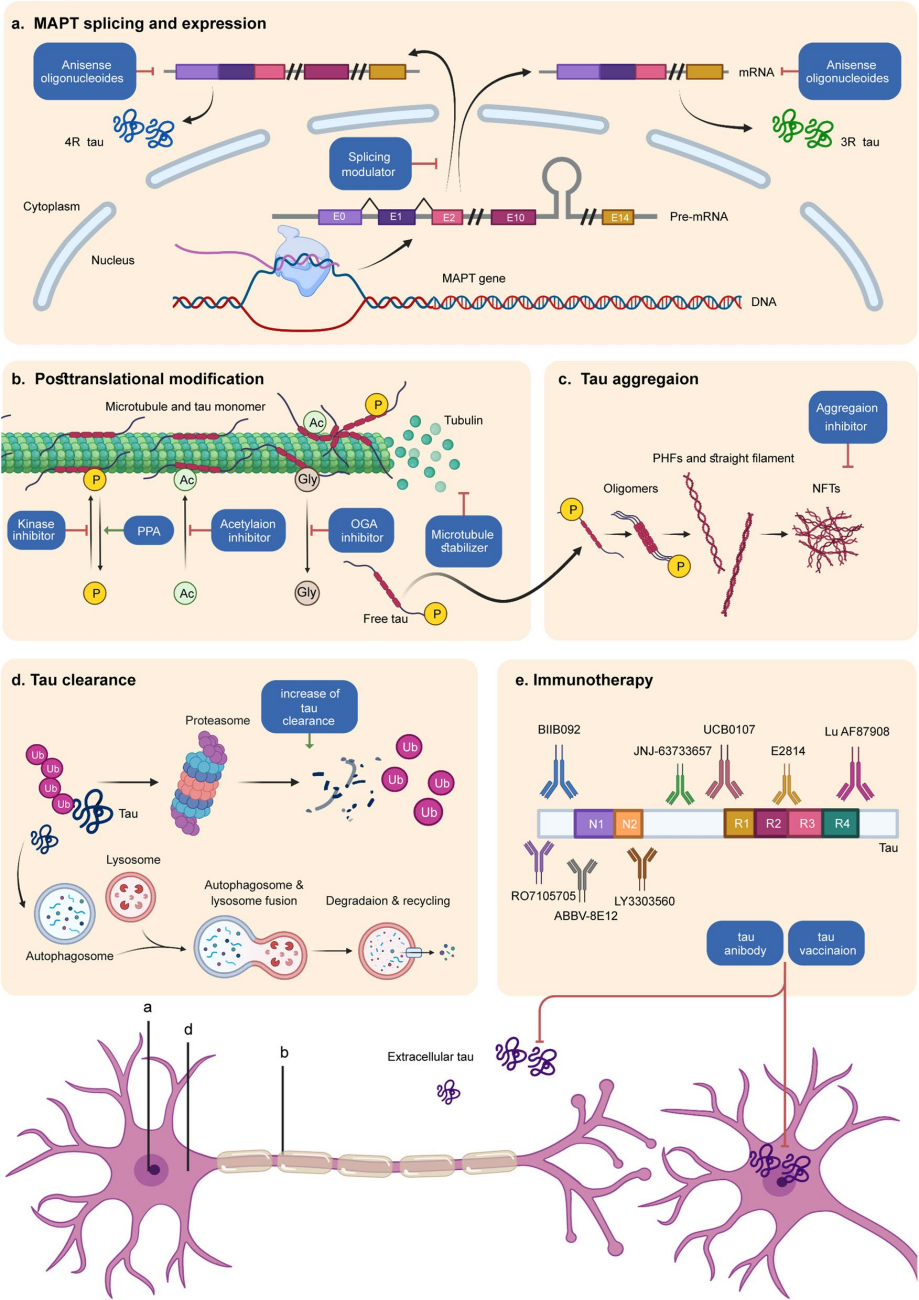
Tau related therapies. Considering the pathogenic pathways existing in tauopathies, potential therapeutic methods include MAPT expression suppression, alternative splicing regulation, microtubule stabilization, post-translational modification adjustment, aggregation inhibition, tau clearance activation, and passive or active immunization. *PPA* phosphatase activator, *P* phosphate, *Ac* acetyl group, *Gly* O-linked N-acetylglucosamine (Gly) residues, *PHF* paired helical filament, *NFTs* neurofibrillary tangles, *Ub* ubiquitination; Created with BioRender.com.

Tau binds to the outer surface of microtubules (MT), prompting the addition of tubulins to MT and stabilizing its structure [166]. In tauopathies, hyperphosphorylation of tau leads to reduced binding affinity, resulting in destabilization of the MT and ensuing axonal deficit and tau pathology [10] (see therapies in Fig. 4).

The first investigated MT stabilizer was paclitaxel, whose initial research was about its anti-tumor property. The poor quality of crossing BBB terminated its further study afterward [167].

Recently, two parallel, double-blind, placebo-controlled randomized clinical trials on TPI-287 (abeotaxane) showed less tolerability among AD patients (more hypersensitivity reactions) than patients with PSP and CBD [168]. Moreover, no relief in symptoms was observed [168].

EpoD (Epothilone D) is a microtubule stabilizing agent. In 2020, a study first treating APP/PS1 mice with EpoD (BMS-241027) concluded that, apart from alleviating axonal/synaptic damage, neurite dystrophy, and neuronal loss, EpoD was also able to reduce amyloid accumulations [169]. However, the phase 1 clinical trial treating AD has been terminated probably due to adverse effect [170].

CNDR-51657, a drug belonging to triazolo-pyrimidines, binds to tubulin at the same site as vinblastine [171]. When administered orally to 9-month female PS19 mice, microtubule defects, axonal dystrophy, as well as tau pathology were significantly relieved [171]. No adverse effects were reported. Moreover, triazolo-pyrimidines have already been investigated as anticancer agents. The advantages of good brain penetration and oral bioavailability have made them promising candidates for MT stabilizers [172].

#### Inhibition of tau aggregation

During disease, tau proteins experience abnormal aggregation, shifting from monomers to NFTs. Therefore, inhibiting aggregation has the potential to alleviate illness. But the pathogenic and toxic tau forms still need further identification, which complicates the strategies targeting tau aggregation.

Methylene blue (MB), a brain penetrable molecule, can inhibit the aggregation of tau in vitro by blocking interaction between tau proteins and influencing the stability of PHFs (paired helical filaments) [173]. But two phase 3 clinical trials of LMTX (TRx0237), a drug derived from MB, demonstrated no significant benefit [174]. Another phase 3 trial showed no differences in prespecified outcomes between two plans (200 mg/day or 8 mg/day) in the bvFTD population [175]. Recently, a randomized, double-blind, placebo-controlled phase 3 trial testing LMTX on AD patients is underway (Table 3). A study explained the poor efficacy of LMTX: although tau aggregation in vitro was attenuated under the treatment of MB, atomic force microscopy discovered an increase in granular tau oligomers, which are more toxic [176], while tau fibrils were reduced [177]. However, the soluble tau oligomers in P301L mice were reduced under the treatment of MB in another study, avoiding cognition decline [178]. Further exploration, such as adopting the BiFC (bimolecular fluorescence complementation) strategy in P301L mice, which realized in vivo investigation of soluble tau oligomers for the first time [178], is needed to elucidate the mechanism.

**Table 3:** Planned or ongoing clinical trials involving tau therapeutics

Drug	Mechanism of action	Disease	Phase	ClinicalTrials.gov identifier
TRx0237	Tau aggregation inhibitor	AD	III	NCT03446001
IONIS MAPTRx	Antisense oligonucleotide	Mild AD	I & II	NCT03186989
NIO752	Antisense oligonucleotide	PSP	I	NCT04539041
Fasudil	Activation of autophagy	PSP, CBD	II	NCT04734379
Rapamycin	Activation of autophagy	MCI, AD	II	NCT04629495
		MCI, AD	I	NCT04200911
ACI-35.030	Tau targeted vaccines	AD	I	NCT04445831
JACI-35.054	Tau targeted vaccines	AD	II	NCT04445831
GV1001	Tau targeted vaccines	Moderate AD	II	NCT03959553
LY3303560	Passive immunotherapy	AD	II	NCT03518073
E2814	Passive immunotherapy	AD	I & II	NCT04971733
UCB0107	Passive immunotherapy	AD	II	NCT04867616
		PSP	I	NCT04658199 NCT04185415
RO7105705	Passive immunotherapy	AD	II	NCT03828747
ABBV-8E12	Passive immunotherapy	AD	II	NCT03712787
Lu AF87908	Passive immunotherapy	AD	I	NCT04149860
JNJ-63733657	Passive immunotherapy	AD Cognitive Dysfunction	II	NCT04619420
LY3372689	O-GlcNAcase enzyme inhibitor	AD	II	NCT05063539
ASN51	O-GlcNAcase enzyme inhibitor	AD	I	NCT04759365
Lithium Carbonate	GSK-3β inhibitor	FTD	II	NCT02862210
		MCI	IV	NCT03185208
Tolfenamic acid	CDK5 and GSK-3β inhibitor	PSP	I/II	NCT04253132
AZP2006	Increases progranulin levels, decreases tau phosphorylation	PSP	II	NCT04008355
Salsalate	Tau acetylation inhibitor	AD	I	NCT03277573
tDCS	NIBS	AD	NA	NCT04855643
rTMS	NIBS	AD MCI	NA	NCT04294888
Sodium Oligomannate Capsules	Anti-inflammation	AD	IV	NCT05058040
JNJ-40346527	Anti-inflammation	AD MCI	I	NCT04121208
VX-745	Anti-inflammation	AD	II	NCT03435861
NE3107	Anti-inflammation	AD	III	NCT04669028
Dasatinib & Quercetin	Senolytic treatment	MCI AD	I & II	NCT04785300
		EOAD MCI	II	NCT04685590
		AD	I & II	NCT04063124
tPBM-2.0	Transcranial Photobiomodulation	AD	II	NCT04784416
Yangxue Qingnao pills	Traditional Chinese herb	AD	II	NCT04780399
Valacyclovir	Anti-virus	AD	II	NCT03282916

*AD* Alzheimer’s disease, *CBD* corticobasal degeneration, *PSP* progressive supranuclear palsy, *MCI* mild cognitive impairment, *FTD* frontotemporal dementia, *NA* Not Applicable, *EOAD* early-onset AD

ACI-3024 is reported to inhibit tau aggregation in cultured neurons and P301L mouse models. Meanwhile, microglia activation and neuronal loss were also reduced [179]. A phase 1 study in healthy volunteers has been done, but no registry entry or published work can be found.

CLR01 has leading effects among molecular tweezers (MTs), which are broad-spectrum protein aggregation inhibitors. In P301S-tau mice, CLR01 was shown to reduce tau aggregation, hyperphosphorylation and oligomerization [180]. No clinical trials have been conducted.

A platform based on recombinant hyperphosphorylated tau realized high-throughput screening for potentially effective compounds. In the results, p-tau aggregation was suppressed by drugs including R-( −)-apomorphine and raloxifene, while benzodiazepines were risk factors for pathology formation [181]. It provides a novel assay for screening effective medicine while identifying risk factors [181].

#### Regulation of post-translational modifications

Phosphorylation, acetylation, glycation, methylation, sumoylation and truncation of tau are post-translational modifications that can occur [34].

Hyperphosphorylated tau tends to self-assemble into PHFs or straight filaments and further becomes oligomers in the cytosol affecting microtubule stabilization. Therefore, drugs targeting related kinases (CDK5, GSK3β, and JNK) and phosphatases (PP2A) have long been under development. Sodium selenate activated PP2A by reducing the inert demethylated catalytic subunit [182]. A 3-month supplement of sodium selenate in THY-Tau22 mice rehabilitated cognition deficits due to neuron damage [182]. A phase 1b clinical trial of sodium selenate on 15 patients with possible bvFTD achieved some therapeutic effects, but a larger placebo-controlled study is required for further confirmation [183].

Acetylation of tau mostly occurs in the fibril-forming core, which facilitates the stacking of β-strand, reducing the solubility of tau [184]. Salsalate works as an anti-inflammatory drug. Meanwhile, the level of tau acetylation decreased at K174 when treating PS19 mice with salsalate, preventing memory loss and hippocampal atrophy [185]. However, an open-label phase 1 clinical trial of salsalate in patients diagnosed as PSP-RS showed no therapeutic effect [186]. No access to the result has been published.

O-GlcNAcylation describes a process adding O-GlcNAc (O-linked β-N-acetylglucosamine) to serine and threonine residues, acting as a protective role against tauopathies. Thus, reducing deglycosylation of tau by inhibiting O-GlcNAcase (OGA) enzyme is a feasible treatment. MK-8719, an OGA inhibitor, reduced tau pathology and retarded brain atrophy in rTg4510 mouse models by improving glycosylation level [187]. Both MK-8719 and ASN120290 received orphan drug designation to treat PSP, but no clinical trial has been reported since then. Two clinical trials on LY3372689 and ASN51 are in progress (Table 3), but no formal preclinical studies are available.

#### Reduction of tau expression

Recently, an innovative antisense oligonucleotides (ASOs) molecule targeting MAPT exon4 successfully downregulated MAPT RNA expression by 96% in cells and reduced the tau protein level by 74% [188]. ASO’s effect has also been proved in several studies [189, 190]. Similar techniques include microRNA (miRNA), small interfering RNA (siRNA) and other transcription inhibitors [10]. A randomized, double-blind, placebo-controlled phase 2 clinical trial of a MAPT-targeting ASOs named Ionis-MAPTRx is conducting (Table 3).

Using AAV-delivered zinc finger protein transcription factors (ZFP-TFs) in mice has also reduced the expression of MAPT at the transcriptional level and alleviated neuronal damage [191]. Moreover, a modified AAV CRISPR-Cas9 construct, which could cross the blood–brain barrier, selectively disrupted the mutated APP allele in the whole brain of mice, resulting in a 6-month relief [192]. Such method may also apply to the MAPT gene.

#### Regulation of exon 10 splicing

The levels of 3R and 4R tau isoforms are approximately equal in healthy adult brains, and disturbance between them is hypothesized to elicit tau pathology [6]. Adjusting the ratio is hence a plausible treatment.

ASOs have long been used to interfere with genetic expression. Apart from degrading targeted mRNA in a DNA-RNA duplex through activated RNase-H, which is mentioned above (9.4 reduction of tau expression), ASOs can also adjust splicing through knocking down mutations disrupting normal splicing pattern, or directly modulating splicing by competing with cis-acting elements and trans-acting proteins [6] like the exon skipping therapy applied in Duchenne Muscular Dystrophy.

Another method which can alter isoform composition is trans-splicing. In htau mouse models, an abnormal ratio between 3 and 4R isoforms was associated with motor coordination impairment. By shifting 3R tau to 4R tau through trans-splicing in early stages, tau isoforms reached an equal proportion without changes in total tau content and motor disorders were relieved [193]. It should be noted that, htau mice obtain nonmutated six tau isoforms like humans, but produce an excess of 3R tau. In humans, therapies tend to reduce exon 10 inclusion and thus reduce pathological 4R tau in some primary tauopathies. An RNA-targeting CRISPR system also corrected the abnormal splicing in neuron models of FTD, providing a supplement to RNA engineering tools [2].

#### Increase of tau clearance

The degradation of pathological proteins has various mechanisms, which mainly include UPS and autophagy-lysosome pathway (ALP), whose function is impeded when tau accumulates in mouse and human brain [194, 195]. Stimulating these pathways is thus a plausible method to cope with tau deposits.

As a highly conservative serine-threonine kinase which decreases autophagic activity, mTOR inactivation through siRNA significantly alleviated tau aggregation in mouse models [196]. The preclinical value of rapamycin, an mTOR kinase inhibitor, is supported by animal experiments. However, rapamycin might only work in the earliest stages of AD [197]. Indeed, two ongoing clinical trials (phase 2 and phase 1) test rapamycin on MCI and early AD patients (Table 3). Besides, by screening a group of small molecules, a study confirmed three competitive mTOR kinase inhibitors, including OSI-027, AZD2014 and AZD8055, all of which demonstrated higher efficacy than rapamycin [194].

Ubiquitination increases protein degradation via lysosome or proteasome. In the P301L mouse model, knockdown of USP13, a de-ubiquinating enzyme, resulted in a lower level of p-tau and increased tau clearance, probably due to autophagy [198]. Possible therapies should target UPS components like USP14, UCHL1, E3 enzyme, PINK1, Parkin and USP30 [199].

Apart from activating autophagy, methods such as directly stimulating lysosome activity and interfering with tau degradation via the endoplasmic reticulum are also viable [200].

#### Tau immunization

Failures in the development of anti β-amyloid immunotherapies elicited the trend of targeting tau pathology through active or passive immunization methods.

##### Vaccinations

Vaccines in active immunization comprise immunogen and various adjuvants. Immunogen options include human full-length tau, PHF-1, phosphorylated tau peptide, truncated and N-terminal regions of tau [201]. IgG was found in the brains of wildtype and transgenic mice treated with AV1980R/A and Advax^CpG^, which are immunogen containing phosphatase activating domains and strong adjuvants respectively [202]. Such treatment also ameliorated tau hyperphosphorylation, especially at Ser396, and improved memory dysfunction [202].

Two vaccines including AADVac1 and ACI35 as the immunogens are under development [201]. DC8E8, which is an efficient antibody targeting microtubule binding domains (MBD) of tau protein, acts as the prototype for AADVac1. A randomized, placebo-controlled, double-blind, parallel-arm, multicenter phase 2 study of AADVac1 on mild AD dementia patients proved its safety but suggested no obvious impact on cognition tests [203].

ACI-35 is a synthetic antigen composed of 16 amino acids (Tau393–408) with phosphorylation at residues S396 and S404 [201]. ACI-35 mainly targets the C-terminal region of tau protein, especially the pS396 epitope and conformational differences, showing a higher affinity for pathologic tau [204]. The employment of this liposome-based vaccine in P301L mice successfully induced antibodies and reduced the soluble tau phosphorylated at S396 in the forebrain and brainstem [205]. A phase Ib/IIa clinical trial is conducted on AD patients in early stages (Table 3).

Antibodies induced by immunogens can reduce misfolded tau protein more efficiently compared with direct antibody treatment [201]. But the immune effect is not sustained and varies between populations, different doses, delivery methods and adjuvants [201]. Possible side effects comprise immune tolerance after persistent stimulation and autoimmune diseases [201].

##### Tau antibodies

Based on the prion-like tau propagation hypothesis, most antibodies tested in clinical trials are IgG4, targeting extracellular tau in transmission [86]. Antibody D (murine equivalent of UCB0107), targeting a central epitope on tau, was shown to effectively block tau transfer between neurons [206]. However, the involvement of exosomes in tau spreading [207] and the fact that intracellular tau constitutes most of the tau pathology, emphasize the necessity of targeting tau inside neurons. A study confirmed the higher efficacy of intracellular targeting in rTg4510 mouse models, as two intrabodies (CP13i and PHF1i, targeting intracellular tau) alleviated tau pathology while scFv (single-chain variable fragments, targeting extracellular tau) showed no therapeutic effect [208].

The therapeutic effect of antibodies is also influenced by epitope selection. Antibodies recognizing central part of tau showed higher efficacy than antibodies targeting amino terminus in cultured neurons and animal models [206]. More specifically, binding MTBD from residues 224–369 showed better results in inhibiting tau transmission [179].

ABBV-8E12 (tilavonemab) and BIIB092 (gosuranemab), which bind tau at the N-terminal side, failed to show efficacy in PSP patients in two phase 2 clinical trials [209, 210]. However, decreased CSF free tau and accumulation of tau in lysosomes of emerging perivascular vesicular astrocytes proved that the binding happens as anticipated [209, 211]. Reasons for futility in clinical trials include difficulties in earlier diagnosis of PSP, especially preclinical stages, noneffective epitopes and extracellular targeting [210]. A phase 2 clinical study testing ABBV-8E12 on AD patients is underway (Table 3). An increasing number of antibodies are under development, shown in Table 3.

#### Immune modulators and other therapeutic approaches

##### Immune modulators

A growing body of literature indicates that chronic activation of the immune system, including glial cells (especially microglia), complement, cytokines, inflammasomes and reactive oxygen species, can contribute to tau pathology exacerbation, apoptosis, and neurodegeneration in AD and primary tauopathies [212–214]. Further evidence suggested that peripheral innate immunity might increase the risk of dementia, while adaptive immunity is linked to a decreased risk of dementia [215]. Microglia are regarded as major players in neuroinflammation. PET analysis in living people revealed that microglia network was associated with tau propagation [216]. They also serve as mediators for some environmental risk factors and lifestyle choices that contribute to dementia [212]. Furthermore, neuroinflammation is potentially associated with disease heterogeneity in pathology, clinical manifestation, and disease severity [217, 218]. However, relationship between immunity and tauopathies is a vast topic, beyond the scope of this review. The interested reader is directed to previous work [212, 219–221]

Sodium Oligomannate Capsules (GV-971) is a marine-derived oligosaccharide, targeting various mechanisms including reconstituting intestinal bacteria which facilitates infiltration of immune cells from the periphery to the brain. In the phase 3 clinical trial just finished, prominent cognitive improvement was observed with no significant adverse effect [222]. A phase 4 trial of GV-971 on Alzheimer’s disease is underway (Table 3).

JNJ-40346527 (JNJ-572), a selective inhibitor of CSF1R, suppressed the proliferation of microglia, neuronal loss, and attenuated behavioral disorders in ME7 prion and P301S mice [223]. A phase 1 study on AD and MCI is in the planning (Table 3).

NE3107 can selectively inhibit inflammatory mediators stimulated by ERK- and NF-κB, without influencing their homeostatic functions [224]. A phase 3 study is planned to examine its safety and efficacy in AD patients (Table 3).

NLRP3 is a kind of pattern recognition receptor. As a part of innate immunity, it combines with ASC to form the NLRP3 inflammasome under stimulation inside microglia [225]. It then activates IL-1β and IL-18 and can lead to cell death (termed “pyroptosis”) [212, 221]. Apart from participating in Aβ cascade inducing tau hyperphosphorylation [225], the NLRP3-ASC axis can be activated by aggregated tau, and in turn exacerbates tau pathology in mice [226]. Lack of NLRP3 inflammasome function ameliorated tau pathology and spatial memory deficits in another study [225]. NT-0796 is reported to selectively inhibited NLRP3 inflammasome, and a phase 1 study on healthy volunteers is underway, testing its safety and tolerability [227], but no registry entry or published work can be found.

What’s more, activating specific inflammatory pathways might also be protective. A Plcγ2-P522R knock-in mouse model showed enhanced phagocytosis in microglia [228]. Another study observed the interaction between astrocytes and microglia through IL-3 and IL-3Rα, which stimulated microglia and ameliorated AD pathology [229].

##### Other approaches

Senescent astrocytes downregulated glutamate and potassium transporters, which led to neuronal death and neurodegeneration [230]. When exposed to extracellular tau, oxidative stress response and activation of inflammasome developed in astrocytes, contributing to cellular senescence and neurodegenerative diseases [231]. Hence, senolytic drugs which clear senescent cells by inducing apoptosis are feasible candidates for dealing with neurodegenerative disease. Clinical trials about dasatinib and quercetin on MCI, AD and EOAD patients are underway (Table 3).

NIBS (non-invasive brain stimulation), encompassing transcranial Magnetic Stimulation (tMS) and transcranial Direct Current Stimulation (tDCS), offers another solution. tMS and tDCS help to improve brain function through exciting pivotal brain areas [232]. A comprehensive summary of published evaluations of the response to NIBS in different neurodegenerative diseases is available in a recent review [232].

## Conclusion

Epidemiology study can also provide insights into disease formation, such as a worth noting decline in incidence of dementia, whose underlying causes might inspire novel disease understanding and treatment.

Manifestation and pathology overlap of tauopathies has led to lack of one-to-one match between symptoms and pathology. Further study on tau ultrastructure and heterogeneity in tau propagation might provide new perspectives for clinicopathological correlations, also aiding the development of specific ligand for tracers and disease-modifying therapies.

Exploration of biomarkers is active nowadays, reflecting the unmet need, especially in non-AD tauopathies and preclinical diagnosis. Emerging works are comparing in vivo binding of biomarkers with neuropathological assessment to confirm their efficacy. Powerful blood-based biomarkers especially plasma p-tau217 and p-tau181, which can more accurately distinguish between AD and FTLD, are recommended to be used in screening and participants selection in clinical trials.

Significant underdiagnosis and misdiagnosis have been found in previous researches, which will mislead our understanding and further direction. Combing genetic information, imaging, and other biomarkers, as well as revising diagnostic criteria and implementing with high quality are important in diagnosis.

Failures to translate results from mouse models into clinical outcomes can be attributed to both preclinical studies and clinical trials, as well as the consistency in interventional stages. Moreover, targeting at early stages of tauopathies is a shared feature in recent trials. And a long-term cohort study comprising large population with detailed outcome and survival information will greatly subserve the discovery of pathogenic factors and novel effective therapeutic targets. Possible development in therapeutics might involve implementation of high-throughput screening, restoring tau physiological role and exploring new targets that highly associated with pathology.

## Data Availability

Not applicable.

## Abbreviations

CSF: Cerebrospinal fluid
PiD: Pick's disease
PSP: Progressive supranuclear palsy
CBD: Corticobasal degeneration
AGD: Argyrophilic grain disease
AD: Alzheimer’s disease
MAPT: Microtubule associated protein tau
CTE: Chronic traumatic encephalopathy
pre-mRNA: Precursor messenger RNA
MTBDs: Microtubule binding domains
FTD: Frontotemporal dementia
bvFTD: Behavioral variant of frontotemporal dementia
CBS: Corticobasal syndrome
FTLD: Frontotemporal lobar degeneration
nfvPPA: Non-fluent variant primary progressive aphasia
svPPA: Semantic-variant primary progressive aphasia
lvPPA: Logopenic variant primary progressive aphasia
PSPS: PSP syndromes
RS: Richardson’s syndrome
PSP-P: PSP with predominant parkinsonism
PSP-CBS: PSP with corticobasal syndrome
PSP-PGF: PSP with progressive gait freezing
PSP-SL: PSP with predominant speech or language disorder
PSP-F: PSP with frontal presentation
PSP-C: PSP with predominant cerebellar ataxia
LOAD: Late onset AD
EOAD: Early onset AD
PCA: Posterior cortical atrophy
bvAD: Behavioral dysexecutive variant AD
NFT: Neurofibrillary tangles
AGs: Argyrophilic grains
GFAs: Granular/fuzzy astrocytes
GGT: Globular glial tauopathy
GAIs: Globular astrocytic inclusions
GOIs: Globular oligodendrocytic inclusions
ARTAG: Aging-related tau astrogliopathy
TSA: Thorn-shaped astrocytes
Aβ: Amyloid beta
PART: Primary age-related tauopathy
PPA: Primary progressive aphasia
LBD: Lewy body dementia
LD: Linkage disequilibrium
GWAS: Genome Wide Association Study
UPS: Ubiquitin proteosomal system
mtDNA: Mitochondria DNA
NfL: Neurofilament light
FA: Fraction anisotropy
WMH: White matter hyperintensities
p-tau217: Tau phosphorylated at threonine 217
p-tau181: Tau phosphorylated at threonine 181
MCI: Mild cognitive impairment
MT: Microtubules
MB: Methylene blue
ASOs: Antisense oligonucleotides
ALP: Autophagy-lysosome pathway
PHFs: Paired helical filaments
NIBS: Non-invasive brain stimulation
tMS: Transcranial Magnetic Stimulation
tDCS: Transcranial Direct Current Stimulation
